# Determinants of Default from Tuberculosis Treatment among Patients with Drug-Susceptible Tuberculosis in Karachi, Pakistan: A Mixed Methods Study

**DOI:** 10.1371/journal.pone.0142384

**Published:** 2015-11-12

**Authors:** Natasha Chida, Zara Ansari, Hamidah Hussain, Maria Jaswal, Stephen Symes, Aamir J. Khan, Shama Mohammed

**Affiliations:** 1 Jay Weiss Institute for Health Equity at Sylvester Comprehensive Cancer Center, University of Miami Miller School of Medicine, Miami, Florida, United States of America; 2 Department of Internal Medicine, Division of Infectious Diseases, Johns Hopkins University School of Medicine, Baltimore, Maryland, United States of America; 3 Interactive Research and Development, Karachi, Sindh, Pakistan; 4 Indus Hospital Research Center, Indus Hospital, Karachi, Sindh, Pakistan; Hospital San Agustín. Aviles. Asturias. Spain, SPAIN

## Abstract

**Purpose:**

Non-adherence to tuberculosis therapy can lead to drug resistance, prolonged infectiousness, and death; therefore, understanding what causes treatment default is important. Pakistan has one of the highest burdens of tuberculosis in the world, yet there have been no qualitative studies in Pakistan that have specifically examined why default occurs. We conducted a mixed methods study at a tuberculosis clinic in Karachi to understand why patients with drug-susceptible tuberculosis default from treatment, and to identify factors associated with default. Patients attending this clinic pick up medications weekly and undergo family-supported directly observed therapy.

**Methods:**

In-depth interviews were administered to 21 patients who had defaulted. We also compared patients who defaulted with those who were cured, had completed, or had failed treatment in 2013.

**Results:**

Qualitative analyses showed the most common reasons for default were the financial burden of treatment, and medication side effects and beliefs. The influence of finances on other causes of default was also prominent, as was concern about the effect of treatment on family members. In quantitative analysis, of 2120 patients, 301 (14.2%) defaulted. Univariate analysis found that male gender (OR: 1.34, 95% CI: 1.04–1.71), being 35–59 years of age (OR: 1.54, 95% CI: 1.14–2.08), or being 60 years of age or older (OR: 1.84, 95% CI: 1.17–2.88) were associated with default. After adjusting for gender, disease site, and patient category, being 35–59 years of age (aOR: 1.49, 95% CI: 1.10–2.03) or 60 years of age or older (aOR: 1.76, 95% CI: 1.12–2.77) were associated with default.

**Conclusions:**

In multivariate analysis age was the only variable associated with default. This lack of identifiable risk factors and our qualitative findings imply that default is complex and often due to extrinsic and medication-related factors. More tolerable medications, improved side effect management, and innovative cost-reduction measures are needed to reduce default from tuberculosis treatment.

## Introduction

Pakistan is one of the highest-burden tuberculosis (TB) countries in the world, with an annual case incidence of 500,000 [1]. The Pakistan National Tuberculosis Program reported universal directly observed therapy (DOT) coverage in the public sector in 2005, and recently began implementing public-private models of care to improve case detection [2,3]. Despite these accomplishments TB continues to be a significant public health threat; Pakistan has become a high-burden multidrug resistant-TB (MDR-TB) country, and in 2013 accounted for 80% of the World Health Organization Eastern Mediterranean Region's MDR-TB burden [1].

Non-adherence to TB therapy can lead to drug resistance, prolonged infectiousness, and death [4]. The Pakistan National Tuberculosis Program estimates the default rate for new cases to be less than 4%, but recent studies have found rates between 7–16% [2,3,5–8]. Default is influenced by factors that vary by geographic location, such as economics and health beliefs; therefore, successful interventions require an awareness of local contexts [9–11]. In addition, understanding the effects of beliefs on treatment behavior is necessary to fully evaluate default [12]. While quantitative research is helpful for identifying risk factors for default, it is unable to completely investigate patients’ reasons for leaving care. The addition of qualitative data is necessary for in-depth evaluations of default in TB programs, yet much of the existing studies on default have not included qualitative analyses [4]. For example, in Pakistan there have been no qualitative studies specifically examining why patients default; three previously published studies did include small sub-groups of defaulters as parts of larger work, but the primary objective of these studies was not to identify reasons for default [13–15].

This study was conducted to determine why patients with drug-susceptible TB in Karachi default from TB treatment, and to identify patient factors associated with default. We achieved these aims by performing a retrospective analysis of the Indus Hospital TB Clinic’s patient database, and by administering in-depth interviews to patients who had previously defaulted.

## Materials and Methods

### Ethical considerations

The institutional review boards (IRB) of Interactive Research and Development, Karachi, Pakistan, and the University of Miami Miller School of Medicine, Miami, United States each approved the study.

### Study setting and population

The study involved participants who had attended the outpatient TB clinic of the Indus Hospital, a free-of-charge 150-bed facility located in a low-income industrial area of Karachi. Over 20 million people live in Karachi; many are migrant workers from varied ethnic backgrounds [6]. Indus Hospital's catchment population consists of 2.5 million people, and approximately 350 patients are seen in the TB clinic each day [16]. The Indus Hospital has one treatment program for patients with drug-susceptible TB and another for patients with drug-resistant TB. This study evaluated patients in the drug-susceptible program. Drug resistance is determined when patients present to clinic for the first time by use of the Gene Xpert^®^ MTB/Rif test; if rifampin resistance is detected, patients undergo drug-susceptibility testing.

Patients with drug-susceptible TB are treated per guidelines from the Pakistan National TB Control Program [17]. Those who are being treated for the first time (Category 1) receive 2 months of isoniazid, rifampin, pyrazinamide, and ethambutol, and 4 months of isoniazid and rifampin. Patients who transfer into the Indus program from another facility while on therapy are also considered Category 1. Patients who are experiencing re-treatment (Category 2) receive of 2 months of isoniazid, rifampin, pyrazinamide, ethambutol, and streptomycin; 1 month of isoniazid, rifampin, pyrazinamide, and ethambutol; and 5 months of isoniazid, rifampin, and ethambutol [17]. Patients pick up medications from the clinic weekly and undergo daily family-supported DOT. The TB clinic keeps a database of contact information for patients who are treated at the clinic. When patients initiate care they are asked if they consent to be contacted for research purposes in the future; those who do not consent cannot be contacted for research.

### Qualitative data collection

A female study member trained in qualitative methods conducted semi-structured in-depth interviews with adult patients (ages 18 and older) who had defaulted from TB treatment. This study member was not involved with clinical care at the Indus Hospital. In-depth interviews were chosen as the method of data collection due to the sensitive and complex nature of reasons for default. The interview focused on reasons for defaulting, but also contained cues related to health experiences, relationships, TB beliefs, and finances (Table 1); the interview was piloted with 5 volunteer clinic patients prior to implementation.

**Table 1:** Examples of in-depth interview questions (English translations).

Please tell me what you know about TB.
Can you tell me why you were unable to complete treatment at Indus?
Can you describe what your overall experience getting treatment at Indus was like?
Now I want you to think back to the time you stopped going to Indus for TB treatment. Tell me what happened and what you did.
Can you describe your relationships with your family after you were diagnosed with TB?
Can you describe your relationships with your neighborhood after you were diagnosed with TB?

TB = tuberculosis

Qualitative study participants were recruited by telephone; using the TB clinic's database of patients, the study member identified patients who had defaulted since the clinic opened in 2007. Out of the 632 patients identified as defaulters in the database between 2007 and 2013, contact information was available for 331. We then sought to employ a maximum variation sampling strategy based on demographic variables, but had difficulty tracing the patients [18]. We subsequently attempted to contact all 331 patients and were able to successfully trace 42 people. Two agreed to participate, while the rest refused. The most common reasons for refusal cited by patients were not believing they ever had TB, feeling better, and being concerned about their community finding out they had been treated for TB.

We subsequently obtained IRB approval to perform telephone interviews and re-called all of the patients who had previously not answered or refused to participate. 19 patients agreed to be interviewed; we therefore conducted 2 in-person and 19 telephone interviews with 10 men and 11 women. During one interview a patient stated she had transferred her care to another facility and had not defaulted; her interview was therefore not analyzed. We intended to use saturation to determine our sample size, but due to our difficulty with tracing patients this was not possible. Of note, the patients we were attempting to contact had defaulted between 2007 and 2013; however, of the 21 who agreed to be interviewed 19 happened to have defaulted in 2013, while 2 had defaulted in 2012.

All interviews were conducted in Urdu (the national language of Pakistan) and took place between June and August of 2014. Both the participants and the study member were alone during the interviews. Prior to each interview the participants were informed of the reason for the study and verbal informed consent was obtained; an IRB-approved oral consent script was used, and documentation of consent was recorded on the script. As the interviews occurred via telephone, written informed consent was not possible. Interviews were audio recorded in their entirety, transcribed verbatim into Urdu, and subsequently translated into English; to maintain anonymity participant names were not included on transcriptions. The median time to complete the interview was approximately 30 minutes.

### Quantitative data collection

The study population included all patients in the drug-susceptible TB program who had a treatment outcome in 2013. Treatment outcomes included the following: “treatment cure,” “treatment failure,” “treatment completion,” “died,” “defaulted,” “change in diagnosis,” or “transferred care to another TB program.” The definitions of these outcomes were derived from World Health Organization and Pakistani guidelines [1,17]. Treatment cure was defined as having confirmed TB and being smear or culture-negative at both the end of treatment and on at least one prior occasion. Due to the definition requiring a smear or culture result at the end of therapy, which is difficult to obtain in patients with extrapulmonary TB, only pulmonary TB patients could be classified as cured. Treatment failure was defined as either a patient with pulmonary TB having a positive smear or culture result at 5 months of treatment or later, or a patient with extrapulmonary TB having a poor clinical response at 5 months of treatment or later. In pulmonary TB patients, treatment completion was defined as completing therapy without evidence of treatment failure, and not having record of a negative smear or culture result at both the end of therapy and on one prior occasion. In extrapulmonary TB patients, treatment completion was defined as successfully completing 6–8 months of treatment. Both treatment cure and treatment completion were considered successful treatment outcomes. Default was defined as starting therapy and having a treatment interruption of 2 or more consecutive months.

Patients who were actively receiving treatment during the study period (that is, had not completed a standard 6–8 month course of therapy and therefore did not have a treatment outcome) were not part of the study population. We limited the analysis to 2013 because this was the year the majority of our qualitative study patients left treatment. Patient demographics and clinical outcomes were abstracted from the TB clinic’s patient database (S1 Dataset). Our primary outcome was default status, which was defined as a treatment interruption of 2 or more consecutive months [1].

### Qualitative data analysis

Analysis was undertaken by the primary author using thematic framework analysis [18]. After a preliminary reading of the transcripts, recurring themes were identified. Inductive codes were developed, and all transcriptions were cataloged according to the defined coding system. The codes were then compiled into larger categories and then overall themes. One additional study member trained in qualitative methods independently reviewed the coding and categories; disagreements were reviewed and resolved by a third study member. The analysis was done manually.

### Quantitative data analysis

The demographic and clinical characteristics of the study population were evaluated. We then performed binary logistic univariate and multivariate regression to assess factors associated with default. Patients in the cohort who defaulted from treatment were compared to patients who had not. Patients who did not default were those who completed a standard 6–8 month course of treatment (regardless of whether they were categorized as treatment cure, treatment completion, or treatment failure). Patients who died, transferred their care to another facility, or had a change in diagnosis were excluded from the univariate and multivariate analysis. Variables in the univariate model that had a *p-*value of 0.2 or less were included in the multivariate model. Odds ratios and 95% confidence intervals for each variable were calculated, and *p*-values of less than 0.05 were considered significant. Data was analyzed using SPSS version 21.

## Results

### Qualitative results

Five themes regarding reasons for default emerged during analysis: the financial burden of treatment, medication side effects and beliefs, TB beliefs, health system effects, and the effect of TB diagnosis and treatment on interpersonal relationships. The themes, categories, and codes are presented in Table 2; quotations are used to illustrate the categories.

**Table 2:** Themes with related categories and inductive codes.

Themes	Categories	Codes
**Financial burden**	Work effects	Missed work to go to clinic and fired or threatened with being fired
		Could not work due to symptoms or side effects
		Family member missed work to go to clinic
	Indirect costs	Cost of transportation to clinic
		Cost of foods and additional medications to decrease side effects
		Treatment costs prevented family from obtaining food, schooling, other needs
**Medication side effects and beliefs**	Side effects	Felt worse on treatment
		Could not perform household duties due to side effects
		Pills were large and painful to swallow
		Decided to try treatment with plans to stop if did not feel better soon
	Medication failed	Side effects meant treatment was not working
		Not feeling better quickly meant treatment was not working
	Medication was harmful	Treatment causes sterility
		Americans changed TB medicine to make patients sterile, like polio vaccine
		Medicine caused more illness than TB did
		Medicine was expired
	Medication success	Felt better so no further treatment required
		Felt better so cost of treatment was an unnecessary expense
		Felt better so not “worth it” to experience side effects
**TB beliefs**	Contingency Plan	Felt better and will return to care if symptoms recur
		Could not afford treatment so will return to care if symptoms become severe
		Will wait until money available to return to treatment
	Curability	TB is curable if one takes medicine
		TB is not curable and is like cancer
		TB is curable but treatment did not work
**Health system effects**	Negative provider interactions	Provider did not listen to concerns
		Provider and clinic staff were rude
	Positive provider interactions	Provider and clinic staff were kind and respectful
		Provider was good but did not understand side effect severity
		Provider was good but unaware medications were harmful
	Healthcare time	Took too long to travel between home and clinic
		Clinic visit took all day and led to worse symptoms
		Clinic visit took family members away from work for too long
	Health system dissatisfaction	All government hospitals and clinics in Pakistan are bad
		The government does not care about TB patients
**Effect of TB diagnosis and treatment on interpersonal relationships**	Supportive home relationships	Family was supportive and helpful
		Family ate less/spent less on themselves so more could be spent on treatment
		Family administered medications and reinforced adherence
	Unsupportive home relationships	Mother-in-law caused illness and was not giving the correct medicines
		Mother-in-law implied having TB decreased masculinity
		Husband forced treatment discontinuation
	Community-based stigma	Friends avoided interaction
		Community members gossiped and avoided interactions
		TB diagnosis must be hidden from the community
	Marriageability	No one will marry someone with TB
		No one will marry the relatives of someone with TB
	Guilt	Unable to contribute to household due to illness
		Cost of treatment was a burden on the household
		Family could suffer social stigma

TB = tuberculosis

Prior to stopping therapy patients carefully considered the perceived benefits and harms of treatment. Their decision to leave care was guided by specific reasons, rather than casual decision-making. Patients reported major, or "primary" reasons for default, then noted "secondary" reasons (which contributed to their default but did not cause it). The most commonly noted primary reasons for default were the financial burden of treatment and medication side effects and beliefs; the most common secondary reason was financial burden. Most patients also described additional problems that made treatment difficult but did not directly contribute to their default; the majority of these problems were related to finances and issues with the health system.

#### Financial burden

Half of the sample reported finances as a primary or secondary reason for default, making it the most common theme referenced overall. Most patients were primarily concerned about the financial burden of the treatment on their families:

"I had no other reason to leave the program but the financial burden it put on my family…my father used to come with me to the doctor, and we would take a rickshaw. The cost would almost be a 100 rupees for one person…200 rupees is a large sum for someone that makes 4000 rupees a month. No one will stop taking medicines unnecessarily. We don’t have a death wish." (female patient)

All of the costs noted by patients were either indirect or direct non-medical costs; for example, the majority of the patients (regardless of gender) were accompanied to clinic, and many reported lost wages for themselves or their accompagnateur due to long clinic waiting and travel times. Overall, half of the sample reported that treatment negatively affected their or their family's work.

Financial concerns also influenced patients’ experiences of other causes of default. Among patients who defaulted due to medication side effects, gastrointestinal symptoms were common. Many bought special foods or over-the counter medications to ease their symptoms, which increased overall costs. In addition, patients often reported having to hire taxis or rickshaws to get to the clinic, rather than taking public transportation; this was because they felt weak from their TB infection, medication side effects, or both:

"I had to take a taxi back and forth (to clinic) because I was weak…Apart from that, I had to pay for the extra medicine I took to keep myself healthy…I had to think twice about coming for treatment because of the extra costs associated with it." (male patient)

#### Medication burden and beliefs

Medication side effects were the most commonly reported primary reason for default. When patients defaulted due to side effects it was because their symptoms were severe and made them feel worse than their TB-related symptoms did:

"I couldn’t sleep at night. Everything made me sick and nauseous…Everything was going wrong…the medication was making me worse.” (male patient)

Some patients thought their side effects indicated the medications had either failed or were harmful. Two male patients stated that both TB medications and the polio vaccine were being given to intentionally cause sterility:

"In the same way that the Americans infiltrated using the polio medicine, they started doing the same with TB medicine…I could not take the risk of not having children." (male patient)

Other patients stopped therapy because they felt better and believed this meant they had received enough treatment, or felt that they could not justify the cost of treatment when their symptoms had improved.

#### TB beliefs

All but 3 of the patients believed TB was curable and thought treatment was beneficial; they often created a contingency plan to return to care if their health deteriorated or if their primary reason for default resolved. In addition, they often realized they might get ill with TB in the future because they had not completed treatment:

"I came to get the medication to push my TB away for a few years so I can set up my life. If I get symptoms again we can take it from there… Right now it was just not smart for me to be a part of the program. I just opted for three months of treatment that I could afford…I got stronger and I was able to start working." (female patient)

The 3 patients who did not believe TB was curable held this conviction prior to becoming ill and had seen family members die of TB. These patients all commented there was no reason to suffer the perceived harms of TB treatment (costs, side effects, etc.), as it would not cure them.

#### Health system effects

Most of the patients noted good health provider interactions; however, despite this they did not accept provider counseling that contradicted their preexisting beliefs about TB:

"The doctor was very nice to me…however it didn’t help take away my fears. I was convinced that I had gotten this (TB) because of my mother-in-law and that it was her fault." (female patient)

Among the 4 patients who defaulted due to negative provider interactions, 3 were dissatisfied with the entire Pakistani health system and believed there was no utility seeking care elsewhere.

#### Effect of TB diagnosis and treatment on interpersonal relationships

The majority of patients hid their diagnoses from their communities due to concerns about community-based stigma, but none stated this was a reason for default. Two men and 3 women were concerned their or their family members' marriageability would decrease if the community found out they had TB:

"Having TB is a horror story. Why would any person want to marry their daughter or son into that kind of an atmosphere? Why would they want to marry me or have a part in my family if I have TB or AIDS for example?" (male patient)

Most patients had supportive home relationships but were highly concerned about the impact of their treatment on their families; the 4 patients who cited guilt as a primary or secondary reason for default all had supportive home relationships. Two patients told their families they were leaving treatment due to side effects or feeling better, when they actually were worried about finances:

"He (husband) used to get less of the other food so he could get more food for me…I always felt guilt…I told my husband the side effects were too much, but the cost was the main problem…" (female patient)

Of the 3 patients who primarily or secondarily defaulted due to unsupportive home relationships, each had a unique experience. One woman stopped treatment because her mother-in-law treated her poorly after her diagnosis, another stopped because her husband demanded it, and one man had no family and felt unmotivated.

### Quantitative results

2120 patients in the drug-susceptible TB program had a treatment outcome in 2013; 66.0% had pulmonary tuberculosis. Successful treatment was achieved by 77.5% of patients, while 14.2% defaulted. The remaining patients died (2.7%), transferred out (2.2%), failed treatment (2.6%) or had a change in diagnosis (0.8%). Table 3 shows the demographic and clinical characteristics of the patients.

**Table 3:** Demographic and clinical characteristics of study population.

Variables	Total (n = 2120) n (%)
**Gender**	
Female	1221 (57.6)
Male	899 (42.4)
**Type of Patient**	
New	1677 (79.1)
Previously Treated	443 (20.9)
**Age**	
0–17	547 (25.8)
18–34	945 (44.6)
35–59	473 (22.3)
60 onwards	155 (7.3)
**Site of TB**	
Pulmonary	1399 (66.0)
Extrapulmonary	721 (34.0)
**Patient Category**	
CAT-1	1802 (85.0)
CAT-2	318 (15.0)
**Treatment Outcome**	
Treatment Completion/Cure	1643 (77.5)
Default	301 (14.2)
Treatment Failure	54 (2.6)
Diagnosis Change	17 (0.8)
Transfer out	47 (2.2)
Died	58 (2.7)
**Stage of Treatment During Default**	
Intensive	189 (62.8)
Continuous	112 (37.2)
**Smear Status at Baseline** *	
Positive	575 (60.4)
Negative	376 (39.6)

*Smear status available for 951 patients with pulmonary TB

TB = tuberculosis; CAT = treatment class; CAT-1 = first treatment with first-line drugs; CAT-2 = retreatment with first-line drugs; Intensive phase = first 2 months of treatment; Continuation phase = 4–6 months of treatment following the first 2 months of treatment

Patients who had died, transferred out, or had a change in diagnosis (totaling 122 patients) were not included in the univariate or multivariate analysis. These analyses were therefore performed on 1998 patients. Our univariate analysis showed that male gender (OR: 1.34, 95% CI: 1.04–1.71), being 35–59 years of age (OR: 1.54, 95% CI: 1.14–2.08), or being 60 years of age or older (OR: 1.84, 95% CI: 1.17–2.88) were significantly associated with default (Table 4).

**Table 4:** Univariate analysis of sociodemographic and clinical variables associated with default.*

Factor	Non-default (n = 1697) *n* (%)	Default (n = 301) *n* (%)	OR (95% CI)	*p-*value
**Gender**				
Female	1002 (86.5)	156 (13.5)	1	
Male	695 (82.7)	145 (17.3)	1.34 (1.04–1.71)	.020
**Type of Patient**				
New	1348 (85.1)	236 (14.9)	1	
Previously Treated	349 (84.3)	65 (15.7)	1.06 (0.79–1.43)	.685
**Age**				
18–34	778 (86.4)	122 (13.6)	1	
0–17	459 (87.9)	63 (12.1)	0.87 (0.63–1.21)	.422
35–59	356 (80.5)	86 (19.5)	1.54 (1.14–2.08)	.005
60 onwards	104 (77.6)	30 (22.4)	1.84 (1.17–2.88)	.008
**Patient Category**				
CAT-1	1453 (85.4)	248 (14.6)	1	
CAT-2	244 (82.2)	53 (17.8)	1.27 (0.92–1.76)	.147
**Site of TB**				
Pulmonary	1107 (84.2)	208 (15.8)	1	
Extrapulmonary	590 (86.4)	93 (13.6)	0.84 (0.64–1.09)	.192

*1998 patients; excludes patients who died, transferred out, or had a change in diagnosis

TB = tuberculosis; CAT = treatment class; CAT-1 = first treatment with first-line drugs; CAT-2 = retreatment with first-line drugs

After adjusting for gender, site of disease, and patient category in the multivariate analysis, being 35–59 years of age was significantly associated with default (aOR: 1.49, 95% CI: 1.10–2.03), as was being 60 years of age or older (aOR: 1.76, 95% CI: 1.12–2.77) (Table 5).

**Table 5:** Multivariate analysis of sociodemographic and clinical variables associated with default.*

Factor	OR (95% CI)	*p-*value
**Gender**		
Female	1	
Male	1.24 (0.97–1.60)	.087
**Age**		
18–34	1	
0–17	0.92 (0.66–1.27)	.604
35–59	1.49 (1.10–2.03)	.009
60 onwards	1.76 (1.12–2.77)	.014
**Patient Category**		
CAT-1	1	
CAT-2	1.18 (0.84–1.64)	.336
**Site of TB**		
Pulmonary	1	
Extrapulmonary	0.92 (0.70–1.21)	.554

*1998 patients; excludes patients who died, transferred out, or had a change in diagnosis

TB = tuberculosis; CAT = treatment class; CAT-1 = first treatment with first-line drugs; CAT-2 = retreatment with first-line drugs

## Discussion

To our knowledge this is the first mixed methods study done in Pakistan to examine why patients default from TB treatment. Our work shows that patient default is most often due to difficulties resulting from treatment (such as cost and side effects), rather than a disinclination to receive therapy. It also reflects the importance of qualitative work when examining reasons for default. For example, the association of finances with default has been previously found, but the degree that finances affected patients' other reasons for default was striking and has not been described in Pakistan [4,11,14,19]. This information would not have been obtained without our patients' descriptions of their experiences. In addition, our qualitative study was the only way for patients to describe the severity of their side effects and how they made treatment unbearable; this highlights the need for the development of more tolerable medications, improved management of side effects, and perhaps early counseling for patients who report significant side effects. Given that clinic visits, medications, and diagnostic testing are free in our setting, our findings also show the significant burden of indirect and direct non-medical costs on patients. Innovative ways to reduce costs are needed; extended clinic timings, transport allowance, and mobile technology such as home video-based DOT may prove helpful in the future [20].

Being 35–59 years of age or 60 years of age or older were the only variables associated with default in our multivariable analysis. Given that in Pakistan employment rates for both men and women peak after age 35, it is possible the effects of TB treatment on patients’ work contributes to defaulting in this age group [21]. Similar effects may also be seen in persons who are working within the household; the number of persons who are married in Pakistan peaks after age 35, and for women this status is often accompanied by an increase in household duties [21]. Being unable to complete household duties due to medication side effects or the cost of TB treatment may negatively affect female patients [22]. With regards to the elderly, while the number of persons who are employed after the age of 60 decreases significantly, prior studies have found that persons over the age of 60 are more likely to experience adverse drug reactions to TB therapy [21,23]. Given that medication side effects were a common reason for default in our study, it is possible this contributed to default among the elderly in our population.

The lack of additional significant risk factors in the quantitative portion our study reflects the impact of external influences on default and the complex nature of treatment behavior. The primary reasons patients cited for treatment non-completion in our qualitative analysis were socioeconomic and treatment-related problems common to many TB patients, regardless of individual characteristics (such as site of TB disease). In addition, uniformly present patient-related risk factors for default across countries and settings have not been found. This is likely due to the effect of local contexts (such as culture, financial systems, and health systems) on treatment behavior and default. For example, prior studies have examined gender as a risk factor for default, but the results have been conflicting; some have found male gender to be associated with default, while others have not [24–29]. In our multivariable analysis gender was not a risk factor for default; this may be due to the fact that in our study both men and women were highly concerned with how indirect treatment costs negatively affected their family members. The effect of the financial burden of TB treatment on family is likely to be similar for men and women, particularly given that TB treatment affected both patients’ and their family members’ work.

Unexpectedly, being previously treated for TB was not a risk factor for default in our quantitative analysis. This may be explained by our qualitative finding that most patients believed TB is curable and that treatment is beneficial; many patients reported wanting to return to treatment when it was more feasible. Perhaps prior treatment experiences do not impact this belief that treatment is efficacious. This conflicts with prior work, which has found that patients who default often believe TB is not curable [14,28,29]. While it is possible this finding reflects recent attempts in Karachi to educate the public about TB, it may also imply a "hierarchy of needs" approach to health [3]. Perhaps patients value the well-being of their families most and make sacrifices to preserve financial security, including leaving treatment. This concept has been noted in India [30].

With regards to our qualitative analysis, given reports from prior studies, we also expected unsupportive relationships to be a prominent cause for default, particularly among women [10,22,30–32]. However, most of our patients reported highly supportive home relationships. Patients’ main source of unsupportive interpersonal relationships was the general community; however, none of the patients stated this as a primary or secondary cause of default. In addition, concerns about marriageability were noted, but unlike prior work in South Asia both genders were worried (rather than women alone) [22]. This again points to the need to examine local contexts when determining why patients default. However, our findings are limited because we did not examine gender differences due to our small sample; further work should explore this.

One of the main limitations of our study is our use of retrospective data to assess variables associated with default; we did not have complete in-depth data available for quantitative analysis due to the nature of the clinic database, and may therefore have missed factors associated with default. We also experienced great difficulty in tracing patients who had defaulted and could not use saturation to determine our sample. This could have resulted in a selection bias. However, while the retrospective nature of the study likely contributed to our inability to trace patients for interview, the default population is often difficult to contact, and small samples are not uncommon [12,13]. This highlights the need for TB programs to identify and engage patients who are beginning to stop therapy before they default, as it is extremely difficult to find them once they leave care. Lastly, while our use of telephone interviews may have decreased the depth of our qualitative work, they have been used with success in the past in other qualitative studies; this effect may therefore not have been significant [33]. Given the difficulty that may occur when attempting to trace patients who have defaulted, and the importance of being able to learn from these patients’ experiences, alternate modes of communication such as telephone interviews may be necessary when patients defer in-person communication.

## Conclusions

TB programs should employ mixed method studies to fully understand default among patients, and use that information to develop strategies to improve TB treatment completion rates. The results of our mixed methods study show that patients’ reasons for default from TB treatment are complex and often due to medication-related issues and extrinsic factors such as financial hardship, rather than an unwillingness to be treated. Being 35–59 years of age or 60 years of age or older were the only variables associated with default in multivariate analysis; this lack of identifiable risk factors may reflect the impact of external influences on default. More tolerable anti-tuberculous medications, improved management of side effects, and innovated ways to reduce treatment-related costs are needed in order to reduce default from TB treatment.

## Supporting Information

S1 Dataset Indus Hospital Outpatient TB Clinic 2013 Patient Database. (XLSX)
